# Mechanical Stress Induces Remodeling of Vascular Networks in Growing Leaves

**DOI:** 10.1371/journal.pcbi.1004819

**Published:** 2016-04-13

**Authors:** Yohai Bar-Sinai, Jean-Daniel Julien, Eran Sharon, Shahaf Armon, Naomi Nakayama, Mokhtar Adda-Bedia, Arezki Boudaoud

**Affiliations:** 1 Department of Chemical Physics, Weizmann Institute of Science, Rehovot, Israel; 2 Racah Institute of Physics, The Hebrew University, Jerusalem, Israel; 3 Laboratoire de Physique Statistique, Ecole Normale Supérieure, CNRS, Université Paris VI, Université Paris VII, Paris, France; 4 Laboratoire de Physique, ENS Lyon, CNRS, UCB Lyon I, Université de Lyon, Lyon, France; 5 Laboratoire Reproduction et Développement des Plantes, Univ Lyon, ENS de Lyon, UCB Lyon 1, CNRS, INRA, Lyon, France; 6 Laboratoire Joliot-Curie, Univ Lyon, ENS de Lyon, CNRS, Lyon, France; 7 Institute of Molecular Plant Sciences, University of Edinburgh, Edinburgh, United Kingdom; 8 Institut Universitaire de France, Paris, France; Department of Computer Science - University of Calgary, CANADA

## Abstract

Differentiation into well-defined patterns and tissue growth are recognized as key processes in organismal development. However, it is unclear whether patterns are passively, homogeneously dilated by growth or whether they remodel during tissue expansion. Leaf vascular networks are well-fitted to investigate this issue, since leaves are approximately two-dimensional and grow manyfold in size. Here we study experimentally and computationally how vein patterns affect growth. We first model the growing vasculature as a network of viscoelastic rods and consider its response to external mechanical stress. We use the so-called texture tensor to quantify the local network geometry and reveal that growth is heterogeneous, resembling non-affine deformations in composite materials. We then apply mechanical forces to growing leaves after veins have differentiated, which respond by anisotropic growth and reorientation of the network in the direction of external stress. External mechanical stress appears to make growth more homogeneous, in contrast with the model with viscoelastic rods. However, we reconcile the model with experimental data by incorporating randomness in rod thickness and a threshold in the rod growth law, making the rods viscoelastoplastic. Altogether, we show that the higher stiffness of veins leads to their reorientation along external forces, along with a reduction in growth heterogeneity. This process may lead to the reinforcement of leaves against mechanical stress. More generally, our work contributes to a framework whereby growth and patterns are coordinated through the differences in mechanical properties between cell types.

## Author Summary

The development of an organism involves a coordination between the differentiation of cells in well-defined spatial patterns and the growth of tissues towards their target shapes. While extensive research has addressed each of these key processes, their coordination has received less attention. In particular, when a pattern has formed and the tissue continues growing, is the pattern passively dilated like a drawing on an inflated balloon, or does the pattern remodel during tissue expansion? We address this question in the context of leaf vasculature and examine the role of mechanics in leaf growth. We model the growing vascular network and identify quantities that compare network growth to background tissue growth. We apply this quantification to mature leaves that are stretched mechanically; we find that vasculature does not dilate passively and that veins reorient in the direction of external forces. This is reminiscent of the reinforcement of bones or of the cytoskeleton so as to resist to mechanical stress. In a developmental context, this might be an essential process to match patterns and growth.

## Introduction

Organismal development relies on both the progressive differentiation of cells according to specific spatial patterns and the growth of tissues and organs towards their target shapes. On the one hand, numerous studies have addressed differentiation mechanisms, leading to a framework where differentiation patterns depend on the establishment of biochemical gradients, see e.g. [1]. On the other hand, it has been shown that simple growth rules can lead to complex morphologies, such as for tumors [2] or ruffled leaves [3–5]. However, the coordination between patterning and growth has received much less attention [6–8]. Are patterns passively stretched by growth like drawings on an inflated rubber balloon, or do patterns remodel during tissue growth? This question is central to the present study.

As growth entails dynamic changes in the structural elements that define shape, such as the cytoskeleton or the extra-cellular matrix, it is essential to address the physical properties of these elements and how these properties are controlled at the cellular level [9–17]. In this framework, cell mechanics would provide a direct link between biochemical activity and growth. Accordingly, the question above can be reformulated as follows. Do the patterns of cell differentiation correspond to patterns of changes in mechanical properties? If so, do changes in mechanical properties predict the geometry of the patterns when the organ reaches its target shape? In addition, what would be the functional role of such changes in the geometry of patterns? Here we use a combination of experiments and mechanical modeling of growth to address these questions within the context of leaf vasculature.

The leaves of dicotylodonous flowering plants and their vasculature provide a fitting context for the study of patterns on growing tissues. Leaves grow manyfold from a sub-millimetric size to several centimeters [18, 19]. They are amenable to genetic [20] or physical manipulation; finally, they can be analyzed quantitatively, being almost two dimensional [21, 22]. Vasculature in dicotyledons is an elaborate reticulated network with striking geometrical and statistical properties, as revealed by advanced mathematical quantification [23–29]. Throughout the leaf’s growth, the network multiplies its size by orders of magnitude while maintaining its crucial structural and functional properties [30, 31]: due to their rigidity [32], veins are the main carriers of mechanical loads in the mature leaf. On the other hand, veins are responsible for the transport of nutrients and water. With this respect, the leaf’s ability to withstand damage of one vein is often ensured by redundancy: the network is reticulated (featuring loops), allowing for alternative routes. Consequently, the venation network, through its topology and geometry, is thought to optimize both its mechanical [33] and transport properties [34]. Finally, vasculature and leaf development appear to be tightly coupled [35, 36].

In many species, the differentiation of ground cells into provascular cells is completed when the leaf is millimetric in size [30]. This process of differentiation is dependent on a biochemical field: the distribution of the phytohormone auxin. The canalization model [37] suggests that the salient features of venation networks are due to instabilities of this field—an initially homogeneous concentration field evolves into a hierarchical network of localized concentrated flow of transported auxin, which eventually becomes the vein system. Canalization has received genetic and molecular support [38–40], while numerical simulations showed that the model accounts for many features of vasculature [41–43]. However, additional hypotheses on transport or on auxin production are needed to account for loops [44, 45]. An alternative model [46] proposed that the mechanical stress field regulates differentiation into provascular cells, motivated by the resemblance between the vascular network seen in leaves and the network of cracks in drying mud, which is known to be created by instabilities of the stress field. Numerical simulations of this model [47, 48] reproduced many features of the network geometry. However the stress field model of differentiation has not received mechanistic support so far.

Here, we do not investigate the process of differentiation of veins, but rather how the vascular network reaches its final geometry. Indeed, after vein formation has ceased the leaf may continue to grow in area by an order of magnitude. Plant growth is driven by the osmotically generated turgor pressure and restrained by cell walls (the extracellular matrix); therefore, mechanical stress can accumulate: for instance, slits made in stems tend to open, indicating that the epidermis is in tension. In leaves, since veins are stiffer than their surrounding environment [32], the vascular network is expected to carry most of the accumulated stress, which might lead to geometrical deformations of the network. This led to the ‘force model’ describing the final geometry of junctions in vasculature [23]: each vein pulls with a force that is proportional to its diameter, and the requirement of local equilibrium at vein junctions leads to a statistical correlation between veins’ diameter and the angles between veins; this correlation was found to hold in the leaves of many cotyledons [23]. More recently, a cell-based mechanical model was developed to describe the time-evolution of the vascular network [49]. The tissue was modeled as a network of viscoelastic cell walls, and vein cells were distinguished from ground cells by their higher rigidity. This yielded realistic venation patterns and reproduced the experimental findings of [23], in line with the force model. However, these studies remain correlative and do not prove that mechanical forces shape the vascular network.

Here, we probe the force model by perturbing mechanically a growing leaf and making predictions about the effect of such a perturbation on the vascular network. We use the texture tensor [50] to quantify this effect; we simulate networks on a tissue that grows anisotropically and predict how leaf vasculature is affected by stretching; we apply external forces to growing leaves after veins have differentiated, and compare observations with predictions.

## Results

### Formulation and geometrical quantifications

**Fig 1:**
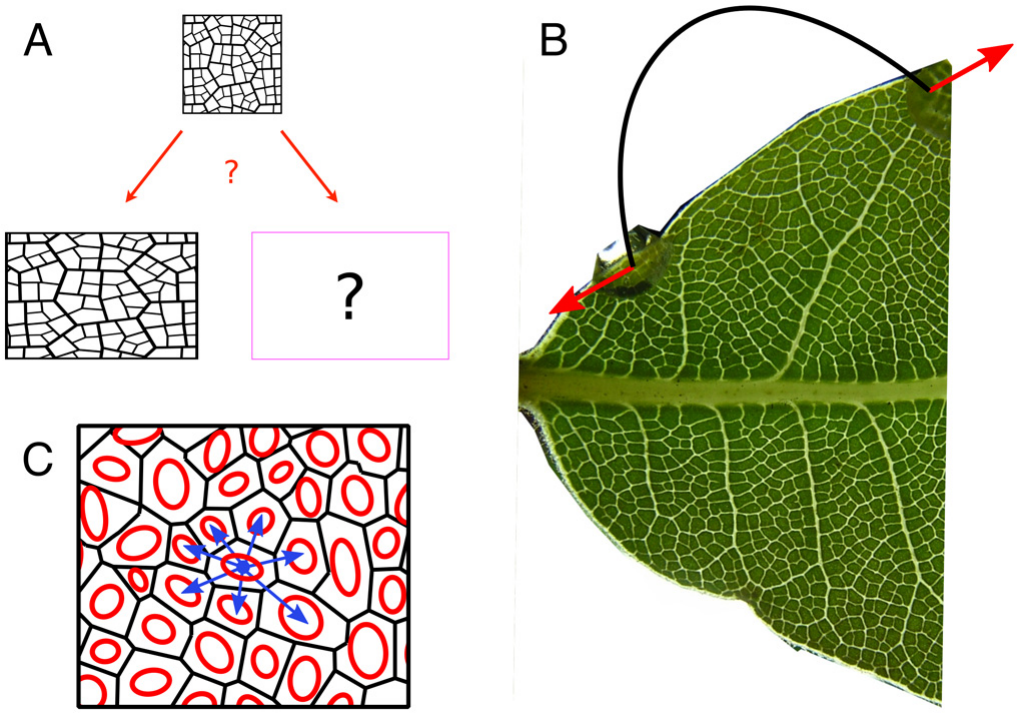
Patterns on a growing tissue: Affine or non-affine behavior? (A) A square-shaped tissue grows anisotropically into a rectangle. Is the pattern passively stretched like a drawing on piece of rubber, or does the pattern geometry influence growth distribution? (B) Typical experiment. A leaf is stretched by applying external forces using a U-shaped steel wire (drawn schematically in black, forces in red). Note the difference in the venation patterns between the stretched and non-stretched sides. (C) Using the texture tensor to quantify vasculature. A part of the vascular network of a leaf, after digitization. Black lines represent veins; the texture tensor of an areole is represented by an ellipse (red) computed from all the vectors connecting its center to the center of neighboring areoles (blue); every ellipse is a representation of the local geometry of the network.

We consider situations in which a leaf grows anisotropically as the result of the application of external forces. More generally, we are interested in the evolution of patterns, here vascular networks, on a growing tissue (Fig 1A): how does the pattern change with growth? Is it merely stretched passively or does its geometry change in a more complex manner? This question is reminiscent of the nature of deformations in elastic solids; in homogeneous solids, elastic deformations are affine, i.e. the local strain is the same as the large-scale strain, whereas in heterogeneous solids, elastic deformations are non-affine, i.e. the local strain differs from large-scale strain [51]. Among biological materials, non-affinity was observed for collagen fibers [52]. Our question therefore amounts to whether growth (an irreversible deformation) is affine or not.

Fig 1B shows a portion of a leaf that was subject to external mechanical stress during two weeks of growth. It is clear qualitatively that the network in the region of the leaf that was grown under tension looks stretched while on the other side it is unaffected. Yet one needs a mathematical method to quantify the strength and orientation of this deformation. Deformation is a tensor, meaning that at each point of the leaf, deformation can occur in many directions: imagine that we draw small circles on the leaf. After growth, each circle will become an ellipse. In order to fully characterize growth, one needs to quantify the orientations and areas of the ellipses, as well as their anisotropies (i.e. how elongated the ellipse is).

Since we are interested in the quantification of the geometrical properties of a network, we use the *texture tensor*. The time-derivative of this tensor was proposed as an equivalent of the elastic strain tensor for the quantification of local deformations in materials with a cellular-like structure [50] and has been used to analyse epithelial morphogenesis [53]. It measures the local geometry of a network, and its time-evolution is a measure of the network’s deformation. Using the texture tensor enables capturing both the averaged, continuum-like deformation as well as the local, discrete deformation of the network’s elements. We give here a qualitative description of the tensor’s definition and properties (see Materials and Methods for details).

The texture tensor, which we denote by **M**, is defined for materials that have a network-like structure, and therefore has a natural application in our case. A network (a *graph*, in mathematical language) is composed of nodes and links that connect between them. In this paper we define the graph by using the areoles (areas surrounded by veins) as the nodes, and we define two areoles as linked (neighbors) if they share a common vein on their boundary. The *local texture tensor* is defined from the vectors linking the center of an areole to the centers of its neighboring areoles, as sketched geometrically in Fig 1C (see Eq 1 in Methods for the exact definition). Thus, the texture tensor contains information not only about the geometry of a single areole, but also about the local topology. In order to obtain properties averaged at the scale of a few areoles, we also define the *averaged texture tensor* from a spatial smoothing (with a constant Gaussian weight) of the local tensor.

Since the texture tensor is a symmetric 2^nd^ order tensor, it describes an ellipse, which is a measure of the local shape of the network. The area of the ellipse (the determinant of texture tensor det(**M**)) quantifies the size of areoles, while anisotropy corresponds to the ratio of the greater axis to smaller axis of the ellipse (ratio of eigenvalues of **M**); note that in this definition anisotropy is always larger than unity.

### A mechanical model of vein reorganisation

#### Simulations

As was mentioned in the introduction, Corson *et al*. [49, 54] developed two numerical models—cell-based and vein-based—that simulate the generation and reorganization of vascular patterns in a growing leaf. The main contribution of these models is that they use only simple mechanical rules for growth regulation and vein creation, yet they quantitatively reproduce the statistics of real vascular networks in dicotyledons.

We modified the vein-based model to include the application of external stress in order to confront the predictions of the model with experimental data. We describe here the main results of the simulations, and provide further details about the simulation in Materials and Methods.

The simulation was conducted as follows. First, a ‘reference’ network was created using Corson’s original cell-based model, so as to create an initial realistic geometry for a portion of a leaf. Then, the network was transformed into an ‘effective’ network, where each vein is replaced by a rod. The background tissue was completely ignored, assuming that veins are much stiffer than ground tissue. This effective 2D network was used as the initial state for the vein-based model.

Each rod was assumed to be viscoelastic and given the mechanical properties of a spring and a dashpot in series. The network was under turgor pressure to make it grow. In addition, external stress was applied to the network in part of the simulations. Following [49], we used periodic boundary conditions in order to avoid boundary effects.

**Fig 2:**
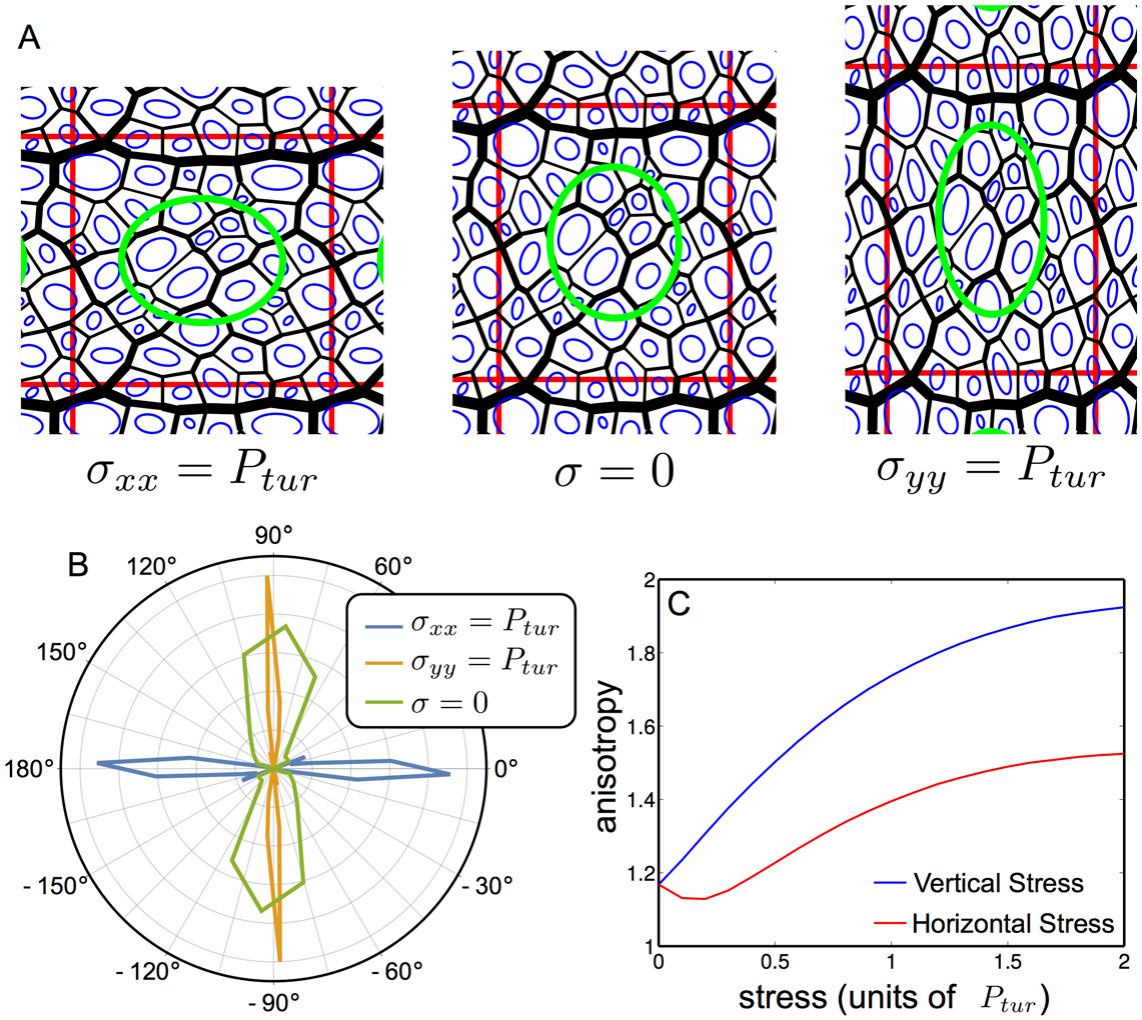
Numerical simulations of a growing network: Anistropy depends on the applied stress. (A) The effect of external stress. Each vein is represented by a black line. The red box is the unit cell with periodic boundary conditions. For visualization, the texture tensor of each areole is shown in the middle of the areole (blue, not to scale), and the averaged texture tensor in the middle of each figure (green, not to scale). The three tissues grew under turgor pressure; in addition, the stress *σ*_*xx*_ along the *x* direction or *σ*_*yy*_ along the *y* direction were set equal to the magnitude of turgor pressure *P*_*tur*_. (B) The distribution of the main orientation (the orientation of the eigenvector associated with the highest eigenvalue) of the texture tensor in the simulation. The distribution is seen to be relatively widely scattered around 90° when no external stress is present (green). When stress is applied, the distribution becomes sharply concentrated around the direction of the external stress (orange and blue). (C) The anisotropy of the texture tensor field averaged over 40 simulations, as a function of the external stress. The stress is measured in units of the turgor pressure, and the anisotropy is measured when the network has doubled its area from the end of the vein creation stage (see Methods).

Fig 2A shows three final states of a simulated vascular network. The same initial network, representing a portion of a leaf, was grown under different external stresses. For visualization, we plot the individual texture tensors of each areole, and the averaged texture tensor of the whole network. The effect of the stress on the tensor’s anisotropy and orientation is evident.

Since the venation network is hierarchical, at any scale of inspection one finds a dominant, thick vein, distinguishable from its surrounding veins. This vein induces a local anisotropy on the network. In our simulations, this was mimicked by the chronological order with which the veins were formed in the vein creation stage. The oldest vein was always the thickest one, and was chosen to be along the *x*-direction. Due to this ‘internal’ anisotropy, even when the external stress is zero the network is not fully isotropic and the orientation is broadly distributed around the mean value of 90° (Fig 2B). This is qualitatively similar to the measurements of a real leaf (S1 Fig). Therefore, one must distinguish two extreme directions in which stress can be applied, which are parallel (*x*-direction) or perpendicular (*y*-direction) to the thick vein.

In Fig 2C we plot the anisotropy and orientation of the simulated texture tensor. As the stress increases, the network becomes less and less isotropic. Also, the orientation of the texture tensor rapidly converges to 0° or 90°, according to the direction of the stress. We also see that when the stress is applied along the *x*−direction it first has to overcome the internal anisotropy, resulting in a decreasing anisotropy for small stresses.

This small drop in anisotropy for small *σ*_*xx*_ can be easily explained by our model, and is consistent with the ‘force model’. Since each vein acts as a rod under tension, and since the thickest vein is along the *x*−direction, the anisotropy makes it easier for the network to stretch perpendicularly to the thick vein. Therefore, in the lack of external stress the network is oriented, roughly, in the *y*−direction (Fig 2B), and stretching in the *x*−direction must overcome this barrier before causing the network to align with the main stress.

#### Does the vascular network grow affinely?

As presented above, the model clearly predicts that external stress affects growth. However, it remains to be shown if the vascular network indeed governs the growth, and does not serve merely as a passive tracer. For instance, the deformation could be a simple dilation along the axis of stretching.

In order to exclude such a dilation, we need to compare the local deformation of an areole and the deformations of surrounding areoles. If the vascular network is passive during growth, we expect individual areoles to deform exactly like their surroundings. Mathematically, this can be performed by comparing the time-evolutions of the local texture tensor and that of the averaged one. In Methods (Eq 2), we define a non-affinity index, *q*, that is equal to 1 only when the areole of interest deforms like its surroundings. *q* quantifies the ratio of the growth rate of the areole to the growth rate of its neighborhood; *q* > 1 (resp. *q* < 1) means that the areole has grown more (resp. less) than its surroundings.

**Fig 3:**
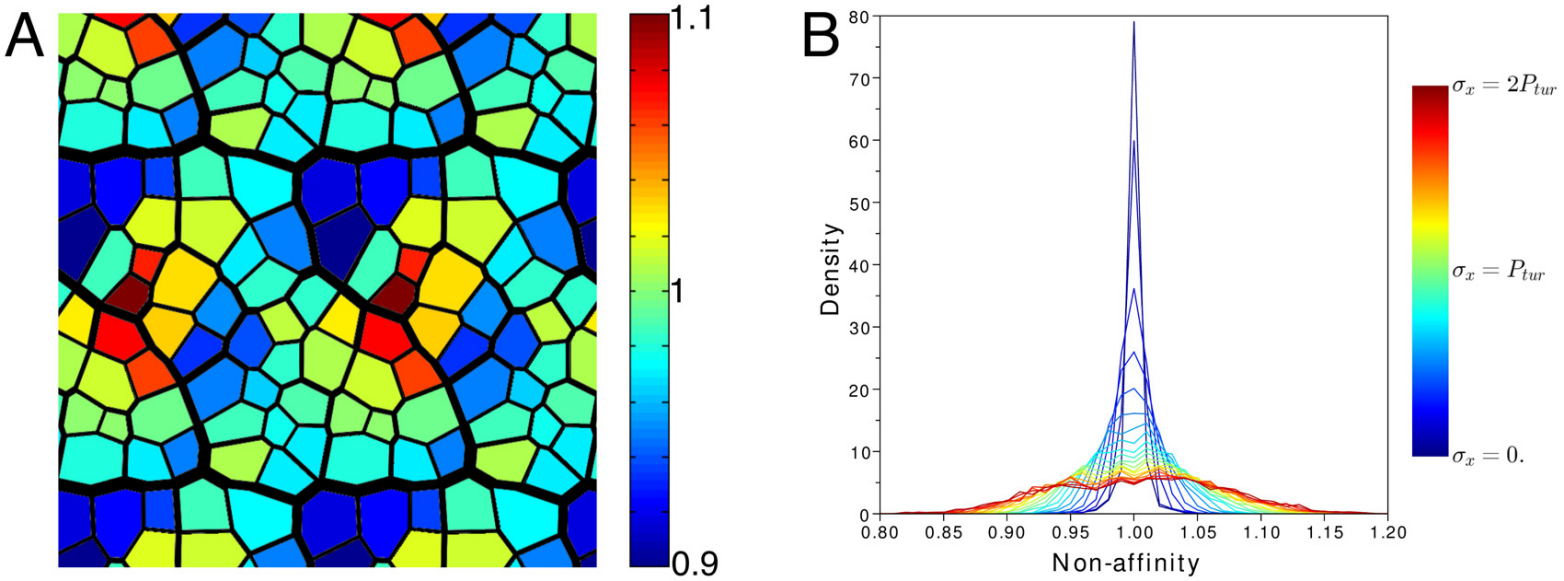
Veins in numerical simulations undergo non-affine deformations. (A) Colormap of the non-affinity index *q* of each areole, for one realization of the numerical simulation with stretching in the *y*−direction (*σ*_*yy*_ = *P*_*tur*_ as in Fig 2); in the case of an affine dilation *q* would be equal to one for all areoles; *q* > 1 (resp. *q* < 1) means that the areole grew more (resp. less) than its neighbourhood. (B) Histogram of *q* over all realizations with stretching in the *x*−direction and for a stress *σ*_*xx*_ ranging from 0 to 2*P*_*tur*_. As in Fig 2, the simulations were stopped when the leaves doubled their area.

In Fig 3A, we plot the spatial distribution of *q* over one realization of the stretched network. *q* is spatially heterogeneous. Furthermore, it appears that *q* differs from 1, as confirmed by its distribution over all numerical realizations (Fig 3B). As the core hypothesis of our model is that veins are stiffer than ground tissues, we predict that the behavior of real leaves grown under external stress will be qualitatively similar.

### Experiments on leaves

We now turn to testing the model in experiments. To do so, we chose to work with leaves in which the vein network has already formed, so as to avoid a direct coupling with differentiation mechanisms. We sought a species such that (i) a large number of veins would improve the statistics and (ii) veins are apparent on photographs to allow for a non-perturbative time-lapse analysis of the geometry of the network. It turned out that bay laurel (*Laurus nobilis*) was appropriate as seen in Fig 1A.

Each leaf was loaded by a U-shaped spring, glued to two points on its edge, typically 3mm apart (Fig 1). The loaded leaf was allowed to grow for 15 days, during which it multiplied its area by about one order of magnitude. The vascular system of the entire leaf was repeatedly photographed. The images were processed and the geometry and topology of the vascular network were extracted. The unstretched half of the leaf was considered as a control. The robust qualitative results were observed in a dozen bay leaves. The detailed mathematical analysis was preformed on three bay leaves. Qualitatively similar results were obtained with tobacco leaves (*Nicotiana benthamiana*, S2 Fig).

#### Large scale effect of external mechanical stress

**Fig 4:**
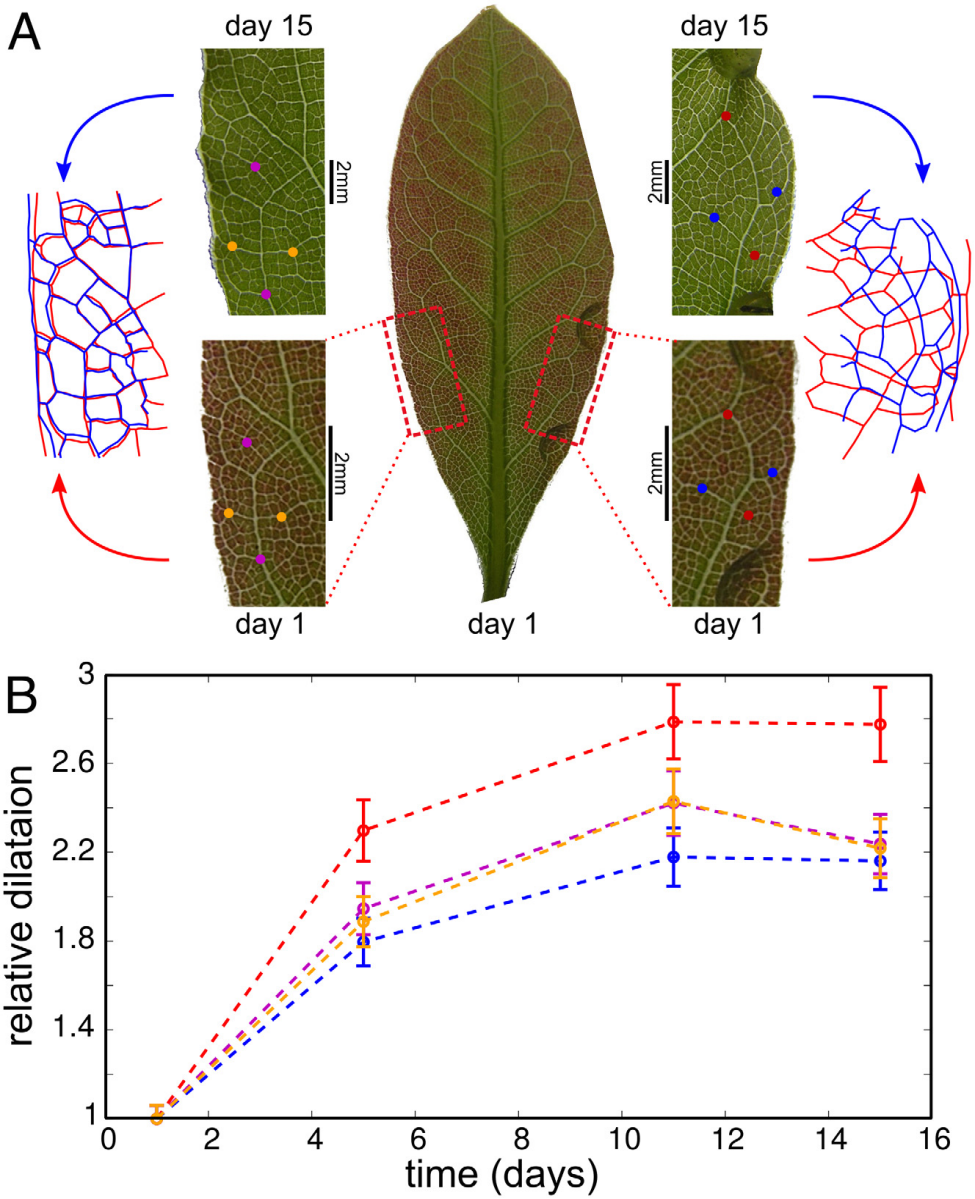
Stretching leaves: Tracing of the venation pattern throughout growth. The transparent drops visible in panel (A) are epoxy glue. The tensile force was applied between these points. The distance between the stretching points is 3.5mm on day 1 and 9.3mm on day 15. In panel (a) we plot the locations of the stretched (right) and freely-grown (left) regions in the leaf on day 1. A close up shows that the freely-grown region grew by isotropic expansion, while the stretched region grew anisotropically (note the different scales for day 1 and day 15). This is confirmed by superimposing the rescaled venation patterns from day 1 (red) and day 15 (blue). (B) Quantification of the large scale anisotropy of growth: The elongation of two perpendicular segments in each region. The endpoints of each segments are marked by colored points in panel (A), and the same color code is used in panel (B). The length of each segment is normalized by its initial value.

Visual examination of the stretched region reveals a clear effect of external stress. Fig 4 shows a closeup of two regions of the same leaf, one that was stretched during growth and another that grew freely. These regions are reproduced on the first day of the experiment, and on day 15. The location of the two regions on the leaf is also shown—they were chosen close to the base of the leaf, symmetrically relatively to the midvein. One clearly sees an effect on the tissue that was grown under tension: while the freely-grown region seems to have grown by mere isotropic expansion, the stretched region shows large deformation of the vascular network along the stretching direction.

We started our analysis by tracing points on the leaf during growth. We arbitrarily selected 4 vein junctions in each of the regions presented in Fig 4A, which were traced for 15 days of growth. Fig 4B shows the distance between two pairs of points as a function of time. While in the non-stretched region both segments have grown by the same ratio, in the stretched area the segment parallel to the stretching direction has significantly grown more than the perpendicular segment. This means that the growth is anisotropic, with the ratio between the principal growth rates of about 1.3. This observation was reproduced in other leaves, and in the same leaf with other junctions traced. Overall, these measurements are a direct evidence of an effect of external mechanical stress on leaf’s growth rates, which is our first main experimental result.

#### Influence of external stress on the texture tensor

**Fig 5:**
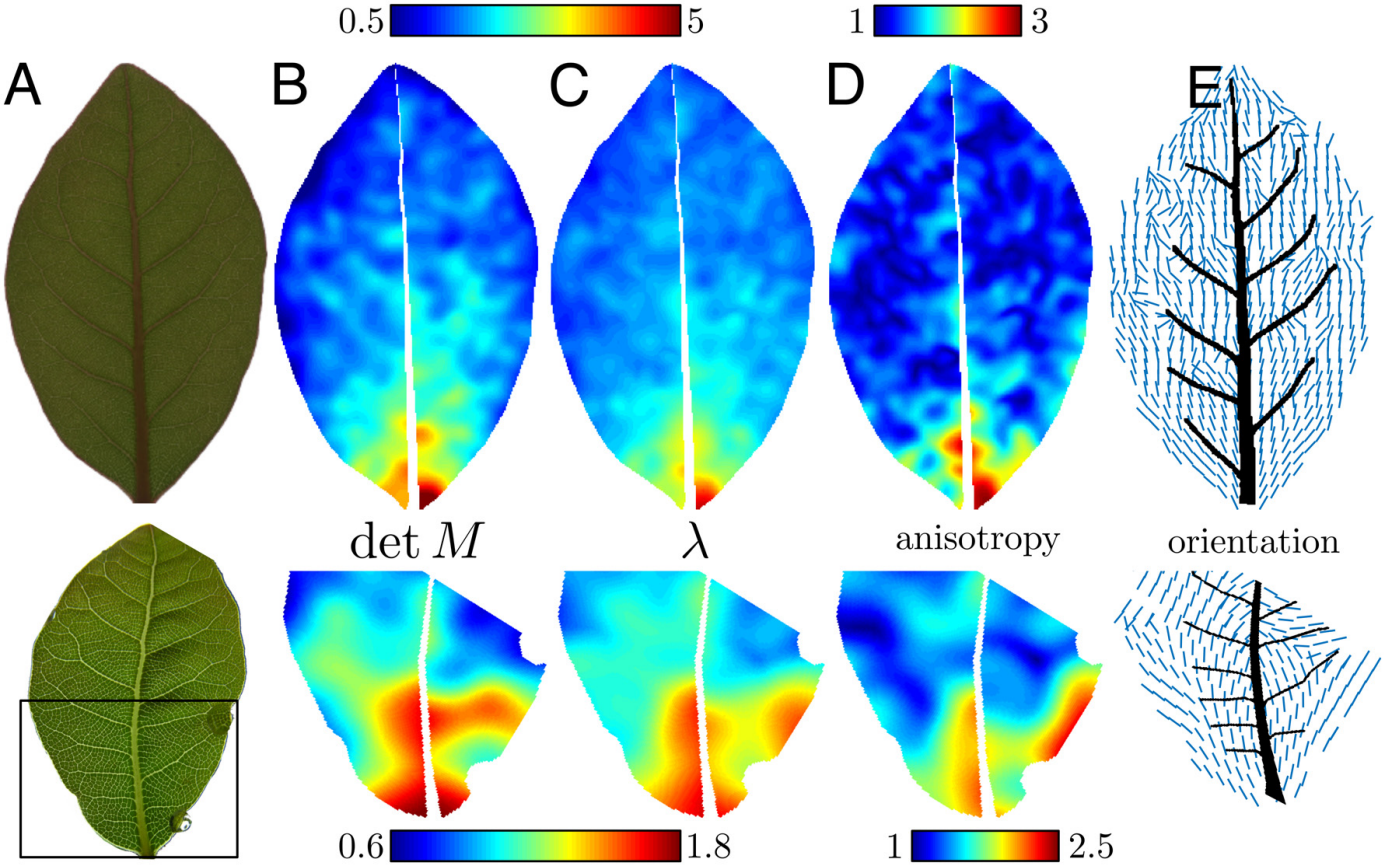
Quantifying the effect of stretching on a leaf. Various scalar quantities defined from the averaged texture tensor *M*, for bay leaves that grew free of external stress (top row), or stretched (bottom row, arrows indicate the force direction). (B,C) The determinant det**M**, which quantifies the size of an areole, and the maximum eigenvalue *λ* of *M*, which quantifies the greater dimension of an areole. These two quantities are normalized by their average value for each leaf. Note the larger areoles at the base of the non-stretched leaf, and in the stretched region of the stretched leaf. *λ* attains its maximum on the edge of the leaf, while the determinant is maximal in the interior. (D,E) The anisotropy and orientation of the texture tensor (or of the corresponding ellipse); larger values of anisotropy imply elongated areoles. The orientation is noisy in regions where the texture tensor is isotropic.

We continued by analyzing the texture tensor in a non stretched bay leaf (Fig 5, top row). Panels (B,C) show the determinant det(**M**) of the texture tensor field and the magnitude of its larger eigenvalue *λ*, which are related to the size and greater dimension of areoles, respectively. Both det(**M**) and *λ* are significantly larger in the vicinity of the leaf’s base—areoles near the base are elongated along the proximo-distal axis. (A similar effect of the leaf’s base on the geometry of veins was reported by Rolland-Lagan *et al* [24].) Further away from the base, one identifies the trace of the midvein, which are more pronounced in the behavior of det(**M**) than in that of *λ*. No significant asymmetry is seen with respect to the midvein, unlike tomato or Arabidopsis [55].

Repeating this for a leaf that was grown under tension for a week (Fig 5, bottom) reveals clear measurable features: both det(**M**) and *λ* are significantly larger in the stretched area. We see that the affected area is easily distinguished form the rest of the leaf. The fact that det(**M**) is larger in the stretched area indicates that growth was enhanced there. However, one sees that the maximal value of *λ* is attained on the margin whereas det(**M**) is maximal in the interior of the leaf. This suggests a relative reduction of growth in the direction perpendicular to the applied forces, a feature that was visible in Fig 4B.

We turn to analyse the orientation and anisotropy of the texture tensor. Panels 5D-E show these fields for both leaves. An obvious feature is that the anisotropy of the freely grown leaf is almost everywhere very close to 1, except near the base. This means that the texture tensor field is almost isotropic, a result which is consistent with the models of [23, 46].

A consequence of this isotropy is that the orientation field is noisy (Fig 5E, top). Still, some important trends are visible: Firstly, on the average the orientation fluctuates around 90°. That is, the texture tensor is, on average, slightly anisotropic and parallel to the midvein. Moreover, a closer examination shows a lateral asymmetry—the orientation in the right side of the leaf fluctuates around 105° while on the left side it fluctuates around 75° (S1 Fig). That is, although the texture tensor measures the network on its smallest scale, it seems to be affected by the orientation of the large veins, which are mirror-symmetric with respect to the midvein. Finally, close to the boundary the orientation is almost parallel to leaf’s margin, as is demonstrated in S1 Fig. This is consistent with the force model: close to a free boundary the normal component of stress vanishes, but the tensile stress along the boundary, due to the turgor pressure, does not. Therefore, we can expect the effect of a free boundary to be similar to that of stretching parallel to the boundary.

In the stretched leaf (Fig 5, bottom), the effect of stretching is striking: one clearly sees that the region between the two stretching points is highly anisotropic, and that the orientation in this region is parallel to applied forces. On the other side of the leaf, where no stress was applied, one sees the same behavior as in the non stretched leaf—small anisotropy. Quantitatively, the ratio in anisotropy between the right and left margins (about 2.5) of the stretched leaf is outside the range of the fluctuations in the control leaf (in the range 1 to 1.5).

All the experimental observations described above clearly indicate that external stress has an effect on growth. In particular, growth is enhanced along the stretching direction (Fig 5C and 5D, bottom, Fig 4). Other effects measured in the non-stretched leaf support a correlation between the network structure and the stress field (Fig 5E, top, S1 Fig).

#### Leaf stretching is not an affine dilation

**Fig 6:**
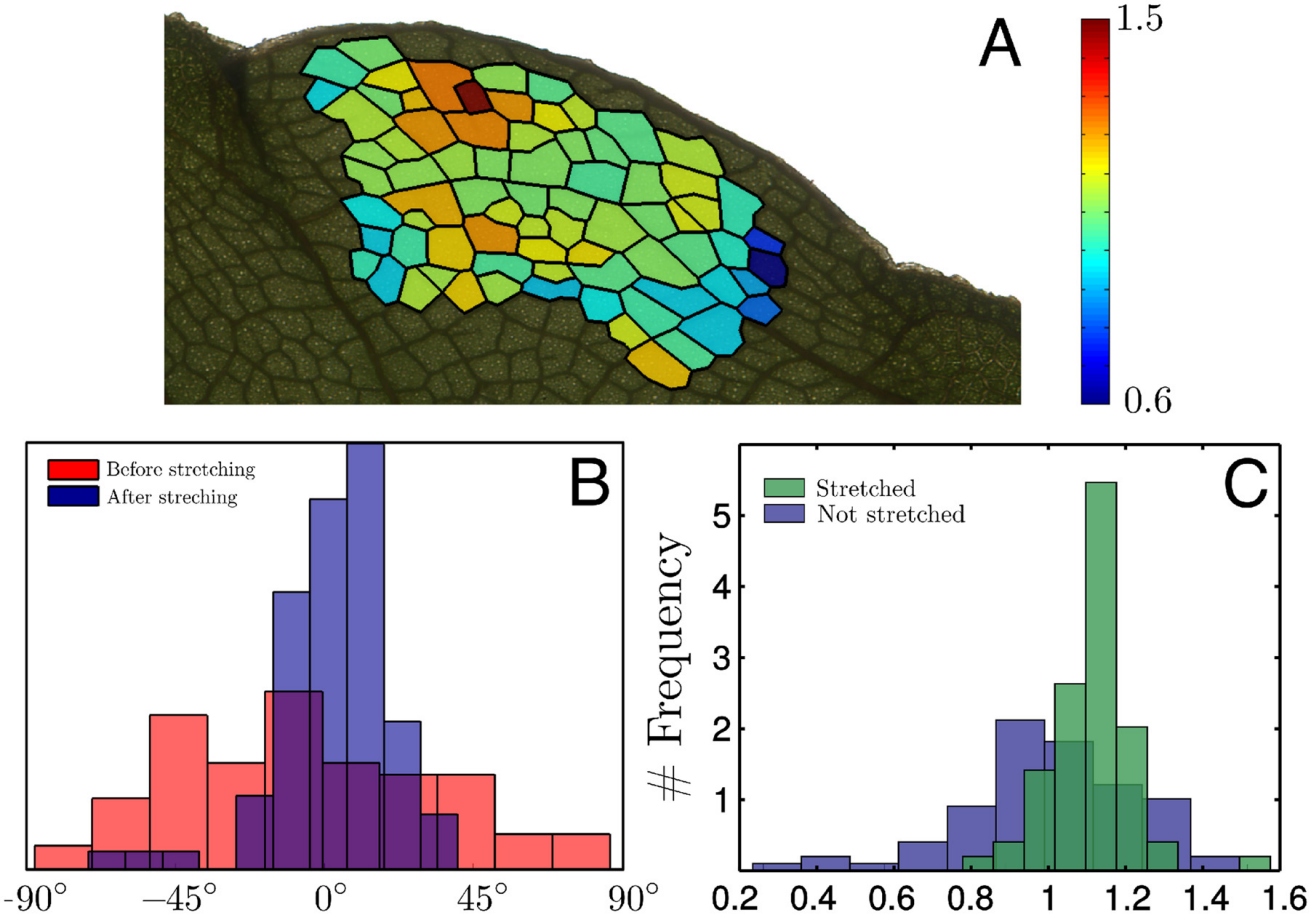
Veins in experiments undergo non-affine deformations. (A) Colormap of the non-affinity index *q* of each areole, after 15 days of external stretching; in the case of an affine deformation *q* would be equal to one for all areoles; *q* > 1 (resp. *q* < 1) means that the areole grew more (resp. less) than its neighbors. (B) Histogram of the orientation of veins before and after stretching showing that veins reorient in the stretching direction. (C) Histogram of *q* in the stretched and unstretched regions.

We now address experimentally the question of the non-affinity of growth. To do so we examine, as in Fig 3, the deviation of the local texture tensor evolution from the averaged one. We compare the areoles before and after 15 days of growth under external stress along the boundary. The final state is shown in Fig 6A. The histograms of vein orientation in Panel (B) show that veins, on average, reorient in the stretching direction. Moreover, the distribution of vein orientations is much more peaked than the case of an affine deformation.

In Fig 6A, the non-affinity index *q* is plotted for individual areoles, where one sees that *q* differs substantially from one, as confirmed by the histogram in panel (C). We note that, in contrast with our simulations (Fig 3B), leaf growth is non-affine even with no external stress. Furthermore, the histogram for the stretched side is narrower than the histogram for the unstretched side. Finally, the average in the stretched side is greater than 1 possibly owing to inhomogeneity of the system due to the organisation of secondary/tertiary veins or to growth distribution.

To conclude, we find that growth is not affine and heterogeneous at the length scale of the areoles, and that stretching reduces this heterogeneity. The latter observation differs from the predictions of simulations, according to which stretching enhances heterogeneity.

### A model with noise and a threshold in the growth law

**Fig 7:**
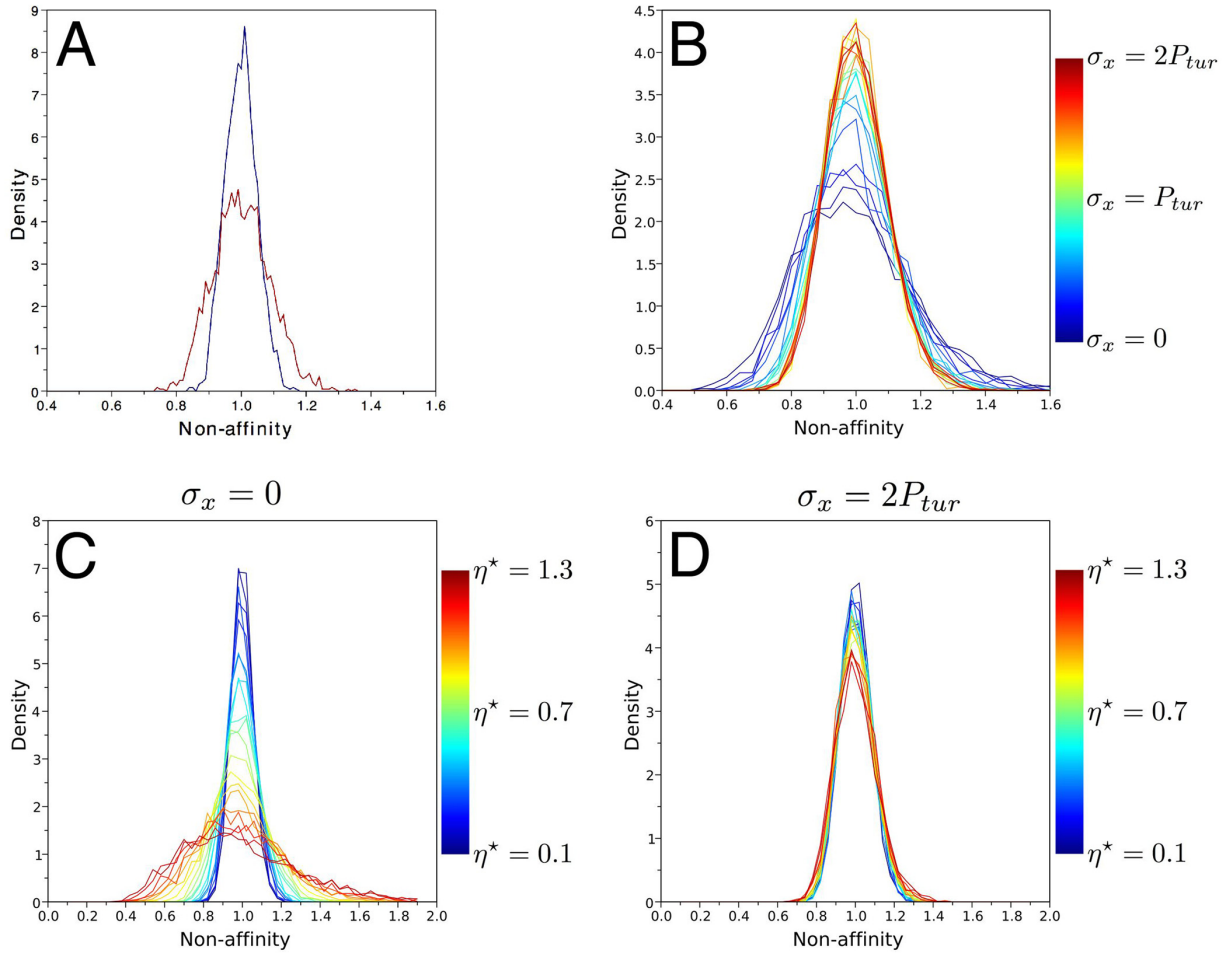
Model with randomness in vein thickness and threshold in the growth law. All panels show the stress-dependent distribution of non-affinity index *q* in all the simulations for a noise amplitude of *r* = 40%. (A) With no growth threshold (*η* = 0) the distribution in the presence of anisotropic stress (red) is wider than without it (blue), in contrast to experimental finding (Fig 6C). (B) When a threshold, *η* = *η*_0_, is introduced, the trend is reversed, and the stressed tissue features a narrower distribution, in agreement with experiments. (C,D) Sensitivity of the results to the threshold; the normalized threshold *η*^⋆^ = *η*/*η*_0_ ranges from 0.1 to 1.3. Panels (C) and (D) correspond, respectively, to *σ*_*x*_ = 0 and *σ*_*x*_ = 2*P*_*tur*_.

We sought to reconcile simulations with experimental data. The broad distribution of non-affinity (*q*) with no stretching (Fig 6C) suggests that the venation network is affected by noise. Indeed, the ‘force model’ was observed to hold only approximately and vein thickness is broadly distributed [23]. We therefore modified the initial state of the rod network by adding noise in rod thickness; each value of thickness was multiplied by a random number uniformly distributed between 1 − *r* and 1 + *r*. We started the simulations from this state and observed non-affine growth. The distribution of *q* is shown in Fig 7A for a noise of *r* = 40% (ratio of standard deviation of thickness to its average), a value that was chosen to match the observed distribution on the unstretched side of the leaf (Fig 6C). Consequently, a frozen noise in vein thickness is sufficient to retrieve observations of non-affinity in unstretched leaves. Finally, non-affinity decreases with vein thickness (S3 Fig) meaning that areoles surrounded by thick veins tend to grow less than their neighborhood in the presence of noise.

However, when we added external stress to the simulation, we found again that the distribution of *q* was broadened (Fig 7A), in contrast with the experimental trend. Thus, we hypothesized that the growth equation, according to which vein elongation rate is proportional to vein tension, was not sufficient to model the system. The narrowing of non-affinity distribution in stretching experiments suggests that high tensions have relatively more effects on vein growth. Accordingly, elongation rate should be a concave function of vein tension. We then recalled that the commonly accepted plant growth law, the Lockhart equation [10, 16, 56], is nonlinear and concave: elongation occurs only above a threshold stress and is then an affine function of stress. We incorporated this into our model and added a threshold to the growth equation (Eq 3 in Methods) in the form

$$\frac{\nu h_{i}}{l_{i}^{0}}\frac{dl_{i}^{0}}{dt}=\max\left(0,T_{i}-h_{i}\eta \right)$$

where *ν* is the effective viscosity, $l_{i}^{0}$ is the rest-length of the *i*-th rod, *T*_*i*_ is the tension in the *i*-th rod, and *η* is the threshold stress for elongation. We first chose the value of *η* equal to *η*_0_ = 6 that corresponds to the average vein stress at the first step of simulations.

We repeated the stretching simulations with this new growth law and we found that the non-affinity (*q*) distribution narrowed under unidirectional external stress (Fig 7B), as in experiments. Moreover the distributions of *q* for *σ*_*x*_ = 0 and *σ*_*x*_ = 2*P*_*tur*_ are quite similar to experimental distributions. In addition, we found that other growth laws (quadratic, with a maximum, with a saturation) yield a broadening of the distribution of *q* when tensile external stress is applied (S4 Fig).

Finally we investigated the robustness of the model by studying the sensitivity of this behavior to the value of the stress threshold. The control parameter was the normalized stress threshold *η*^⋆^ = *η*/*η*_0_, *η*^⋆^ = 1 corresponding to our first successful trial. We did not consider values of *η*^⋆^ < 0.1 as the model converges to the initial model with no threshold, as well as *η*^⋆^ > 1.3 as the tissue stopped growing because the tension in all rods remains below the threshold. With no external stress, increasing the threshold broadens the non-affinity distribution, as shown in Fig 7C. With high external stress (*σ* = 2*P*_*tur*_), the distribution of *q* is insensitive to *α* as the behavior of the system is then dominated by external stress. Importantly, the distribution of *q* is broader with no stress, as in experiments, when *η*^⋆^ > 0.7.

We therefore conclude that our model reproduces experimental observations when including noise and a Lockhart-like growth law as long as the mean vein tension is not much higher than the threshold tension.

## Discussion

In order to address the coordination between patterns and growth during the course of organismal development, we studied the response of leaf vasculature to external stress. More specifically, we investigated whether leaf vasculature is merely dilated by growth, like a drawing on a balloon that is inflated, or whether growth is non-affine. We combined the simulations of a two-dimensional mechanical model of vasculature with the experimental manipulation of leaves in which veins have formed.

The main assumption of the model was that veins are much stiffer than ground tissues. The application of anisotropic external mechanical stress resulted in elongated areoles in simulations; on average, the long axis of the areole corresponded to the direction of the maximal stress. To quantify this effect, we used the texture tensor, which is a good measure of the local geometry of the network. We found that, overall, the anisotropy of the texture tensor increased with the level of stress.

We then used the texture tensor to quantify experiments on leaves. While measuring the geometry of leaves before stress application, we retrieved known features of leaf geometry and growth. On the one hand, areoles are bigger and more anisotropic near the base of the leaf, which might be ascribed to an enhanced growth, reflecting the gradient in maturation along the leaf axis that occurs at the later stages of leaf development [22, 57]. On the other hand, the anisotropy of areoles follows the left-right symmetry of the leaf and its local geometry; the major axis of areoles parallels secondary veins and the margin, while on average, it is aligned with the leaf axis. When external mechanical stress is applied to the leaf, areoles become elongated in the direction of the largest stress, as in simulations.

Nevertheless, the elongation of areoles might only be a passive consequence of the largest growth in the direction of external force. To test this possibility, we used the texture tensor to quantify non-affinity. We found that, both in simulations and experiments, the local change in texture tensor differed from the average change, demonstrating that growth is heterogeneous and non-affine. However the simulated distributions differed in behavior from experimental distributions. Therefore we modified the model by incorporating noise in thickness and a threshold in the growth law. Both this model and experiments featured heterogeneity in growth, which was reduced upon external stretching.

A threshold in the growth law was introduced by Lockhart [56] to describe experimental data showing that a minimum turgor pressure was needed for growth to occur. This model is well-supported in situations with growth along one axis, as in single cells or in cylindrical plant organs [10, 16]. Our results further support this model in a two-dimensional setting.

While we cannot exclude more complex hypotheses involving biochemical feedbacks, it is more parsimonious to ascribe our observations to the vein mechanics that induce non-affine growth. It is still left to find out whether they can be explained by a simple viscoelastic behavior of the veins as implemented in the model, or whether they also involve a more sophisticated regulation process. In the former case, it suffices that veins have a specific ‘mechanical identity’, being stiffer than ground tissues, as is obvious in mature leaves [32]. If additional regulation existed, it might be manifested, for example, by softening of cell walls in correlation with stress, or by preferential thickening of veins that carry higher loads. However none of the growth laws that we tried yields results that agreed with observations, except the one with a threshold.

In this context, one should note that the effect of stress exists only when the leaf is growing: we did not observe any measurable effect when we applied stress to mature leaves that do not grow in area, or to areas that stopped growing within a growing leaf. Pursuing this direction, we wondered whether non-affinity was also applicable to the earlier stages of leaf development. We thus examined leaf primordia in *Arabidopsis thaliana*. While this species does not fulfill the requirements stated above for an experimental investigation of the effects of external forces, many molecular and genetic resources are available, such as a reporter for early vascular identity (*pVH1::GUS*, see Materials and Methods). Using this reporter, we visualized veins in dissected leaf primordia (S5 Fig); the midvein appears to be smooth and almost straight before tertiary veins have formed, while at later stages it features kinks at the junctions with secondary veins (S5 Fig). This observation indicates that the shape of the midvein does not change according to a simple dilation of the leaf but rather that growth is inhomogeneous and influenced by the local geometry of vasculature, consistently with our observations on older leaves. This might seem at odds with the work in [8], showing that growth fields in early leaves can be accounted for by the affine dilation of an initial polarity pattern, but this work considered younger primordia: tertiary veins appear only at the end of the periods monitored there.

To conclude, we showed that, in leaves in which the vasculature has formed, veins reorient in the direction of applied external forces, and that the geometry of the midvein suggests that this also applies to leaves in which vasculature is differentiating. It would be interesting to investigate whether this is relevant to vasculature in animals [58, 59], to veins in insect wings, or more generally to netted patterns of differentiation in other growing tissues. Our results further support the force model [23, 49], according to which most of the mechanical load is carried by the veins (or equivalently, that the veins are stiffer) and that the tension in each vein is proportional to its thickness. Our results may imply that the network changes so as to become reinforced in the direction of the main stress. This reinforcement would be reminiscent of Wolff’s law according to which bone remodels so as to resist changes in mechanical stress, or of the reorientation of cortical microtubules in plant cells according to the direction of highest stress [60–62]. Similarly to these studies, applying external stress helped us identify a response to internal stress, which can result from differential growth. However, we note that the reduction in growth heterogeneity with higher anisotropy of mechanical stress differs from work in the shoot apex showing that the reorientation of cortical microtubules according to external forces induces growth heterogeneity at the cell scale [63]. These mechanisms operating at different scales might reflect a form of homeostasis, in which the tissue becomes anisotropically stiffer so as to resist the effect of external forces, and which would also underlie the coordination between patterning and tissue growth.

## Methods

### Texture tensor

Deformations and growth are associated with the mathematical concept of a second rank tensorial field. The texture tensor was proposed in [50] for quantifying local geometry in materials with cellular-like geometry; the time-derivative of the texture tensor allows the quantification of geometry. In this paper we define a graph by taking the areoles to be the graph’s nodes, and defining two areoles as connected if they share a common vein. One can also define the dual graph, whose nodes are the vein junctions, linked by veins. This method gives similar, yet more noisy, results. For each node *i*, located at ${\vec{r}}_{i}$, the *local texture tensor* is defined as

$$M_{i}=\frac{1}{N}\sum_{k}\left({\vec{r}}_{k}-{\vec{r}}_{i}\right)⊗\left({\vec{r}}_{k}-{\vec{r}}_{i}\right)$$ (1)

where the summation runs over all the neighbors if the site *i*, *N* is the number of ${\vec{r}}_{i}$’s neighbors, and ⊗ denotes the standard 2D tensor product, defined by ${\left(\vec{u}⊗\vec{v}\right)}^{\alpha \beta }=u^{\alpha }v^{\beta }$ where *α*, *β* are Cartesian coordinate indices. In experiments, the texture tensor is undefined for areoles that are on the boundary of the leaf. The process is sketched geometrically in Fig 1C.

This gives the *local texture tensor*, which is defined only on the graph’s nodes ${\vec{r}}_{i}$. To get the *averaged texture tensor*, $M(\vec{r})$, which is a continuous field defined everywhere on the leaf, the local tensor is averaged over the whole leaf with a Gaussian weight centered at $\vec{r}$. The width of the Gaussian, *σ*, is chosen so that the area *πσ*^2^ is 30 times the mean area of an areole. This value of 30 was determined to reveal general trends, but the results were insensitive to the width of the Gaussian, in a range around this value. When the averaged tensor is used for an areole, we take its value $M({\vec{r}}_{i})$ at the areole center ${\vec{r}}_{i}$.

At each point the texture tensor field describes an ellipse, which is a measure of the local shape of the network. The determinant measures the area of the ellipse and the directions of the tensor’s eigenvectors indicate the ellipse’s orientation. The eigenvalues are the lengths of the ellipse’s axes, and we define the *anisotropy* of the tensor to be the ratio of the larger to the smaller axis. Note that by definition the anisotropy is always larger than 1.

### Non-affinity index

At each time step, we compare the local texture tensor **M**_*i*_(*t*) of each areole *i* to the averaged texture tensor $M({\vec{r}}_{i},t)$, using the ratio of their determinants. If dilation were locally homogeneous, or equivalently if growth were affine, this ratio would be independent of time, because the geometry of the network would be the same up to a magnification factor. Therefore we define the non-affinity index of areole *i* between time *t*_1_ and *t*_2_ as

$$q_{i}=\frac{\det M_{i}\left(t_{2}\right)}{\det M({\vec{r}}_{i},t_{2})}/\frac{\det M_{i}\left(t_{1}\right)}{\det M({\vec{r}}_{i},t_{1})}.$$ (2)

If the network was affinely dilated, then *q* = 1 in all areoles because the ratio of det**M**_*i*_(*t*) to $\det M({\vec{r}}_{i},t)$ would be time-independent. The deviation of *q* from unity quantifies the differences between the local and averaged behavior of the areole. An equivalent index can be defined using areal growth [64], see S6 Fig, that is related to the coefficient of variability of growth introduced in [63].

### Simulations

As described earlier, the simulations were built upon the work of F. Corson *et al* [49, 54]. We give here a brief description of the model, and refer the reader to [49] for details. Corson’s model consists of an array of interconnected viscoelastic rods, modeling the cell walls, in a two-dimensional periodic boundary condition space. The difference between cell walls of the background tissue and cell walls of the vascular tissue is manifested in their elastic properties—vein cell walls are stiffer when oriented with the direction of the vein.

The simulations were divided to two stages: In the creation stage, a ‘reference’ network was created using Corson’s model, which yields networks statistically similar to real venation networks [49]. In the reorganization stage, creation of new veins was arrested, and the network was transformed into an ‘effective’ network, where each vein was replaced by a viscoelastic rod, with the same thickness *h*_*i*_ and rest length $l_{i}^{0}$, given by those of the vein that it represents. The background tissue was erased. The process is shown in S7 Fig. In order to have an ideal initial configuration, we further optimized vein thickness so that the tension carried by each vein is proportional to its thickness.

The linear viscoelastic behavior of the rods is manifested in the change of the rods’ rest length, given by

$$T_{i}=\mu h_{i}\left(\frac{l_{i}}{l_{i}^{0}}-1\right)=\frac{\nu h_{i}}{l_{i}^{0}}\;\frac{dl_{i}^{0}}{dt}$$ (3)

where *T*_*i*_ is the tension in the *i*-th rod, *μ* is the vein’s Young modulus, *ν* is its viscosity, and $l_{i},l_{i}^{0}$ are its length and rest-length, correspondingly. The network was grown in quasi-static conditions, at each time step minimizing the elastic energy of the network, which is given by

$$E_{el}=\sum_{i\in \text{veins}}\frac{1}{2}\mu h_{i}{\left(\frac{l_{i}}{l_{i}^{0}}-1\right)}^{2}-P_{tur}S-E^{ani}\;,$$ (4)

where is the turgor pressure, and *S* is the total area of the network. While Corson’s model was restricted to isotropic stress, we introduced an external stress by an anisotropic term in the energy:

$$E^{ani}=WH\epsilon_{ij}\sigma_{ij}=H_{0}\left(W-W_{0}\right)\sigma_{xx}+W_{0}\left(H-H_{0}\right)\sigma_{yy}$$ (5)

where *W*, *H*, *W*_0_, *H*_0_ are the network’s width and height, reference width and reference hight, respectively. The definition of the reference width and hight is done by calculating the equilibrium configuration of the network without the term Eq (5) in the energy equation.

The rod model was implemented in C. The energy is minimized according to the BFGS algorithm using the NLopt library. The system of ordinary differential equations is solved using the GNU Scientific Library. All parameters were set to 1 except for *μ* = 300. Thus the typical strain was around 0.02 in the initial conditions. The energy was minimized every Δ*t* = 10^−5^.

### Stretching experiments

The experimental set-up consists of attaching a U-shaped steel wire stretcher to a growing leaf, using epoxy glue. After polymerization, the glue was attached to the leaves’ trichomes. The leaves showed no pathologic behavior in response to the glue as could be checked in leaves where two glue drops were deposited with no spring.

The applied stress is of same order of magnitude as the turgor pressure. We present a rough estimation: The order of magnitude of the stress is *σ* ≈ *F*/*S* where *F* ≈ 1 grams ≈10*N* and *S* is the surface of the stretched area, perpendicular to the applied force. We estimate the affected area to be about 1 cm wide. The leaf thickness is of the order of 1mm. Therefore we have

$$\sigma =\frac{F}{S}\approx \frac{10N}{10^{-3}m10^{-2}m}=10^{6}Pa=10\text{atm}$$

which is of the same order of magnitude as the turgor pressure. In the numerical model, the external stress was in the range 0 < *σ*/*P*_*tur*_ < 2.

In order to quantify the vascular network, the leaf was photographed using a commercial digital camera (Nikon CoolPix 8800VR), with strong back-light. The different optical properties of the vascular network allow it to be easily distinguishable from the rest of the leaf. The vascular network was then extracted from the image either by semi-automated image processing methods (written in Matlab) or manually.

During several repetitions we noticed that the effect of external force is much more pronounced when stretching close to the base of the leaf, which might be ascribed to the fact that in later stages of development, growth is concentrated near the base of the leaf [22, 57].

### Arabidopsis material

We used *Arabidopis thaliana* Col-0 transgenic plants expressing *β*-glucuronidase under the control of the promoter of *VASCULAR HIGHWAY 1* (*pVH1::GUS*), an early vascular marker [65]. Plants were grown in soil in long day (16hrs day/8hrs night) conditions and at 20–22°C and harvested two weeks after sowing. The plants were stained for GUS activity in 10mM sodium phosphate buffer (pH 7), 10mM EDTA, 0.1% Triton X-100, 0.5g/L X-glucuronic acid, and 10mM ferri- and ferro-cyanide for 24hrs at 37°C and subsequently cleared in 70%-100% ethanol for 2 days. Leaves were dissected and mounted in 70% glycerol and pictured with a Zeiss Axiophoto microscope and Axiovision software.

## Supporting Information

S1 Fig Orientation of areoles in mature leaves. (A) The distribution of orientations of the local texture tensor in an unstretched leaf (black). Note that the distribution is widely distributed around 90°, which is the direction of the mid-vein. The data is also partitioned into the left (blue) and right (red) sides of the mid-vein, which are widely distributed around the direction of the secondary veins. (B) The orientation of the local texture tensor along the margin, as a function of the local direction of the margin.
(PDF)

S2 Fig Stretching tobacco leaves. Scans of tobacco leaves stretched as in Fig 1 for 7 days and then cleared in lactic acid for 24 hours. The blue arrows indicate the forces applied. (A) A whole leaf and magnification of equivalent regions along the left and right margins. A deformed vein network can be observed in the proximal part of the stretched region. (B) Magnification of a stretched region. (C) Magnification of an unstretched region.
(PDF)

S3 FigNon-affinity and vein width.The non-affinity index *q* of an areole plotted as a function of the average thickness of veins surrounding the areole, without (A,C,E) or with (B,D,F) external stress; the middle line stands for the average value of *q* over all simulations and the top and bottom lines show the average plus or minus one standard deviation. At the top of each subfigure, the growth law specifies the relative growth rate $1/l_{i}^{0}\;dl_{i}^{0}/dt$ as a function of effective viscosity *ν*, tension in the vein *T*, vein thickness *h*_*i*_ and law parameter *η*^⋆^. (A,B) Linear growth law and no noise. (C,D) Linear growth law and initial noise on vein thickness (amplitude *r* = 40%). (E,F) Growth law with a threshold and initial noise on vein thickness (amplitude *r* = 40%).(PDF)

S4 FigModel with randomness in vein thickness and various growth laws.All panels show the stress-dependent distribution of non-affinity index *q* in all the simulations for a noise amplitude of *r* = 40% and external stress that increases from *σ*_*x*_ = 0 (blue) to the maximum value *σ*_*x*_ = 2*P*_*tur*_ (red). The growth law is shown at the top of each subfigure, specifying the relative growth rate $1/l_{i}^{0}\;dl_{i}^{0}/dt$ as a function of effective viscosity *ν*, tension in the vein *T*, vein thickness *h*_*i*_ and law parameter *η*^⋆^. (A) With a growth threshold. (B) Quadratic. (C) With a maximum. (D) With a saturation. Only the law with a threshold leads to a narrowing of non-affinity under external stress.(PDF)

S5 Fig Relevance to young, differentiating leaves. Veins are marked by the early provascular reporter *pVH1::GUS*. We classify leaves according to whether they have tertiary veins (older leaves, e.g. L3) or not (younger leaves, e.g. L1 and L2). The shape of the midvein in younger leaves is smooth and straight (59 out of 62 leaves) while for older leaves, it is generally kinked at junctions with secondary veins (37 out of 51 leaves). Examples of kinked and unkinked junctions are marked with red and green arrows, respectively. All leaves are at the same scale, L1 being ∼200*μ*m in length.
(PDF)

S6 FigAlternative definition of the affinity index using areal growth.Consider areole *i* that grows from area *S*_*i*_(*t*_1_) at time *t*_1_ to *S*_*i*_(*t*_2_) at time *t*_2_. The local relative areal growth rate is defined as *a*_*i*_ = ln[*S*_*i*_(*t*_2_)/*S*_*i*_(*t*_1_)]/(*t*_2_ − *t*_1_). The averaged relative areal growth rate is defined from the local one exactly as the averaged texture tensor is defined from the local one. Finally the non-affinity index of areole *i* is $a_{i}/a\left({\vec{r}}_{i}\right)$. No qualitative changes are seen when replotting Fig 3B using this new definition of the affinity tensor: histograms are shown shown over all realizations with stretching in the *x*−direction and for a stress *σ*_*xx*_ ranging from 0 to 2*P*_*tur*_.(PDF)

S7 Fig The mapping from the vein creation stage to the reorganization stage. In the left picture each vein and areole contains hundreds of cell walls which are smaller than the resolution of the image. In the right picture each vein is mapped to a viscoelastic rod, and background tissue is completely ignored.
(PDF)
