# Supplementary figures and images for: Mechanical Stress Induces Remodeling of Vascular Networks in Growing Leaves
Source: PLoS Comput Biol. 2016 Apr 13;12(4):e1004819. doi: 10.1371/journal.pcbi.1004819 (PMC4830508; doi:10.1371/journal.pcbi.1004819)

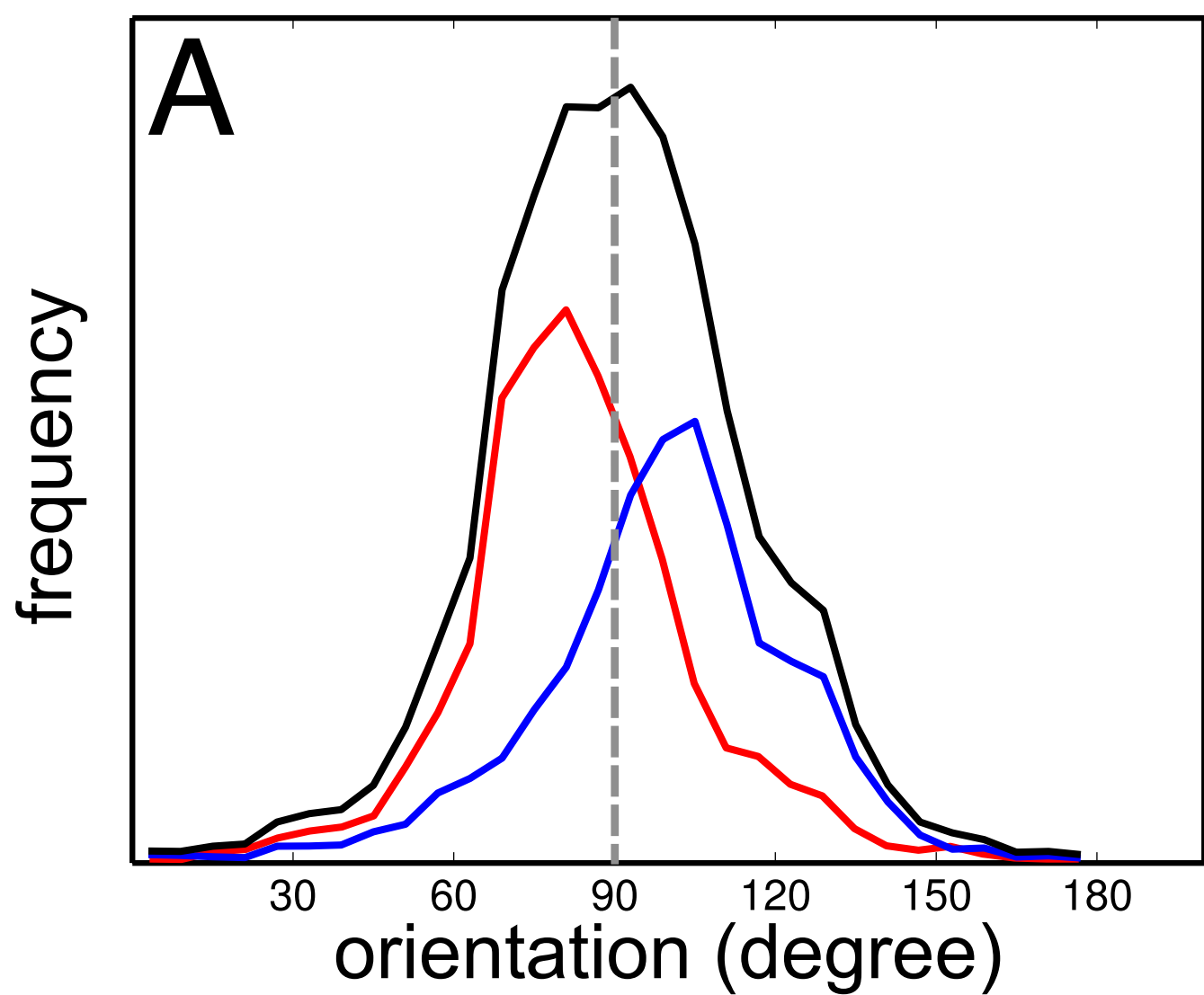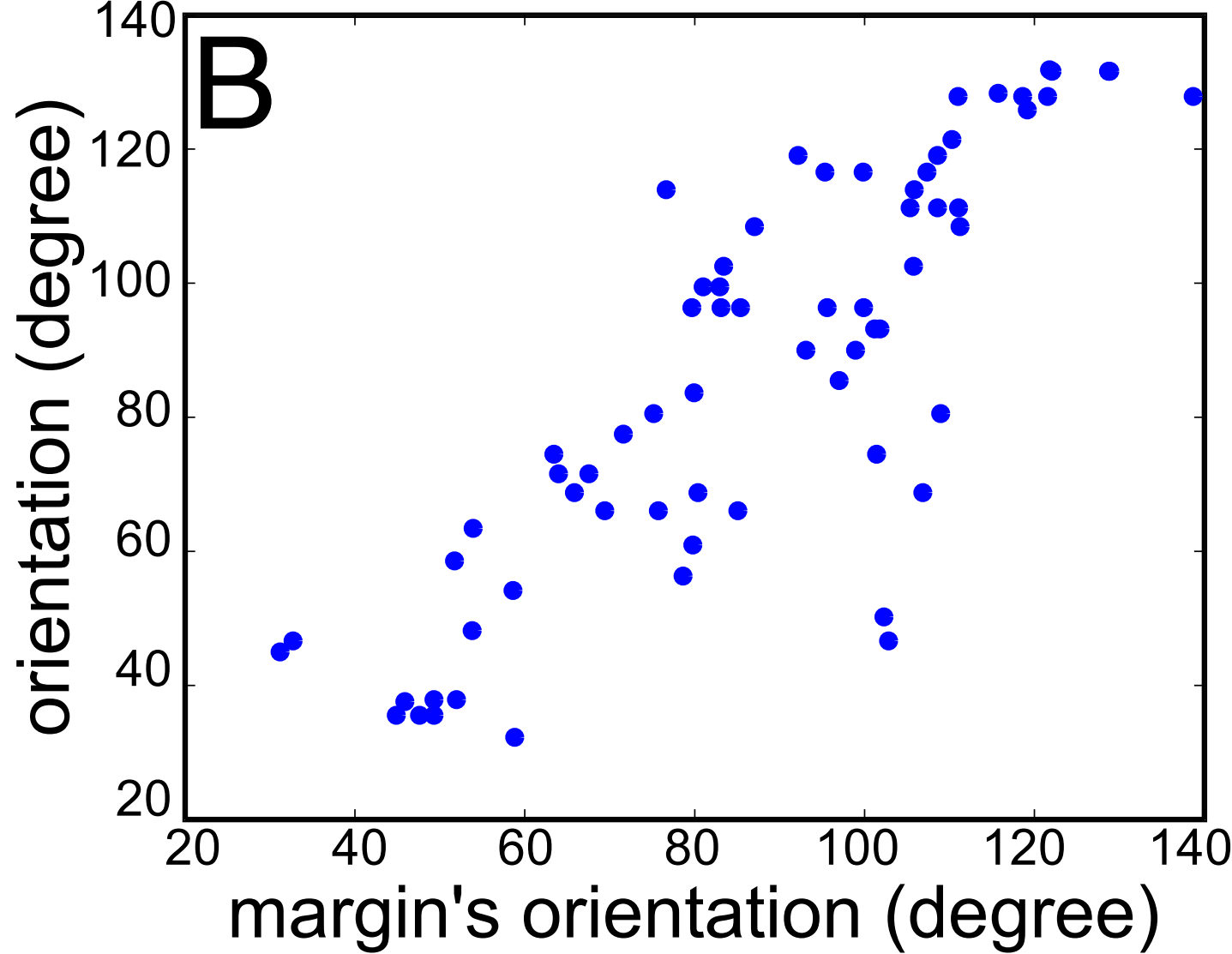

Supplement: S1 Fig — (A) The distribution of orientations of the local texture tensor in an unstretched leaf (black). Note that the distribution is widely distributed around 90°, which is the direction of the mid-vein. The data is also partitioned into the left (blue) and right (red) sides of the mid-vein, which are widely distributed around the direction of the secondary veins. (B) The orientation of the local texture tensor along the margin, as a function of the local direction of the margin. (PDF) [file pcbi.1004819.s001.pdf]

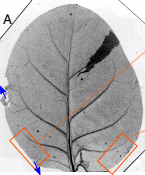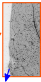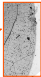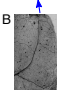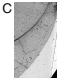

Supplement: S2 Fig — Scans of tobacco leaves stretched as in Fig 1 for 7 days and then cleared in lactic acid for 24 hours. The blue arrows indicate the forces applied. (A) A whole leaf and magnification of equivalent regions along the left and right margins. A deformed vein network can be observed in the proximal part of the stretched region. (B) Magnification of a stretched region. (C) Magnification of an unstretched region. (PDF) [file pcbi.1004819.s002.pdf]

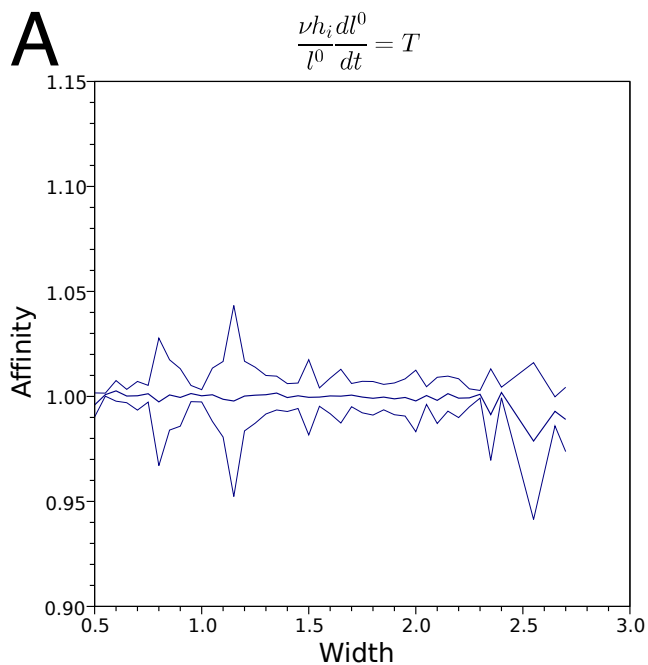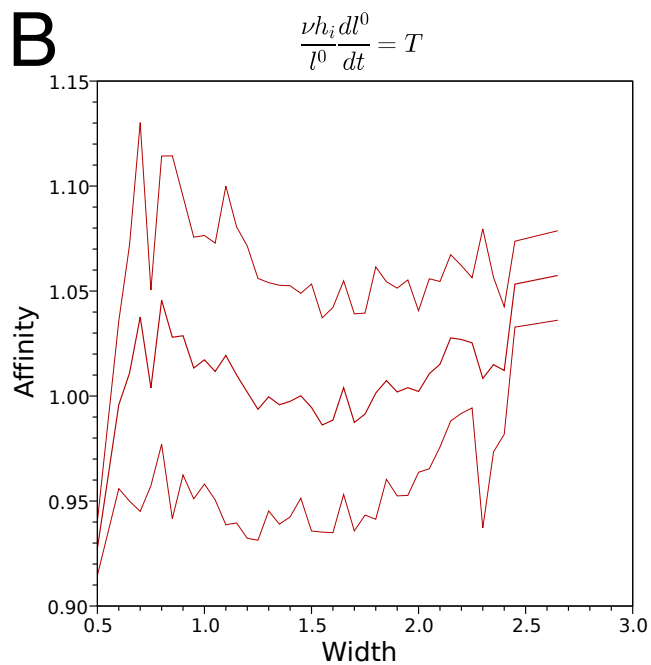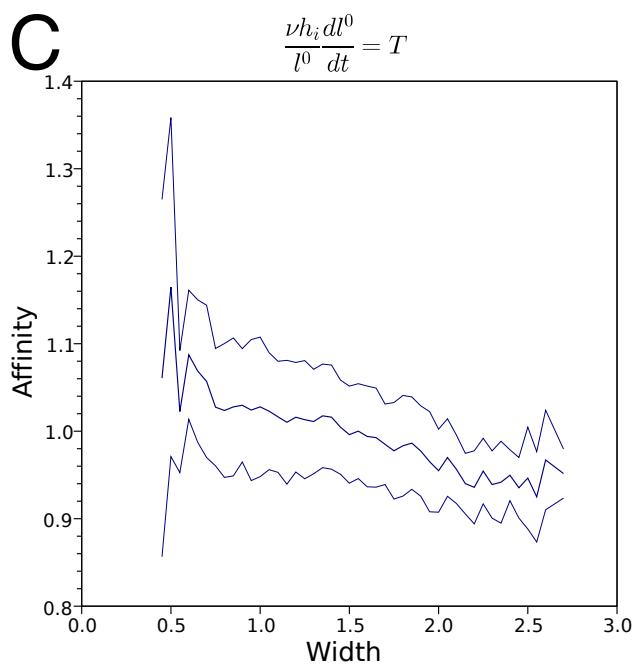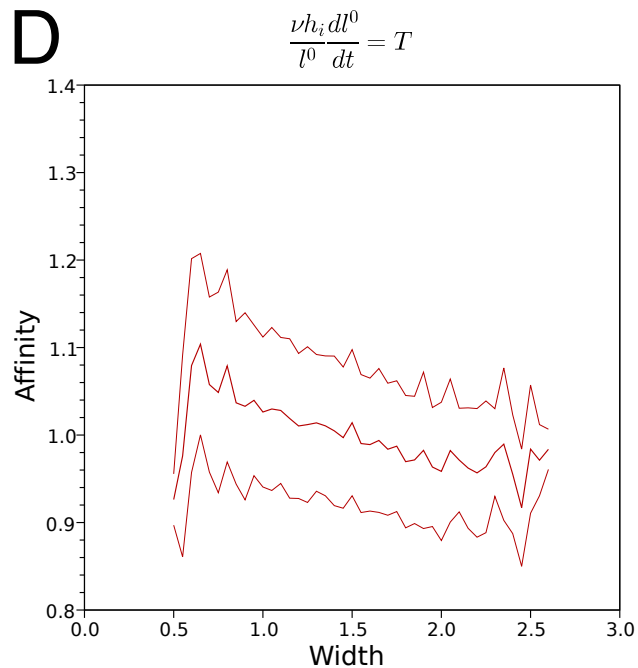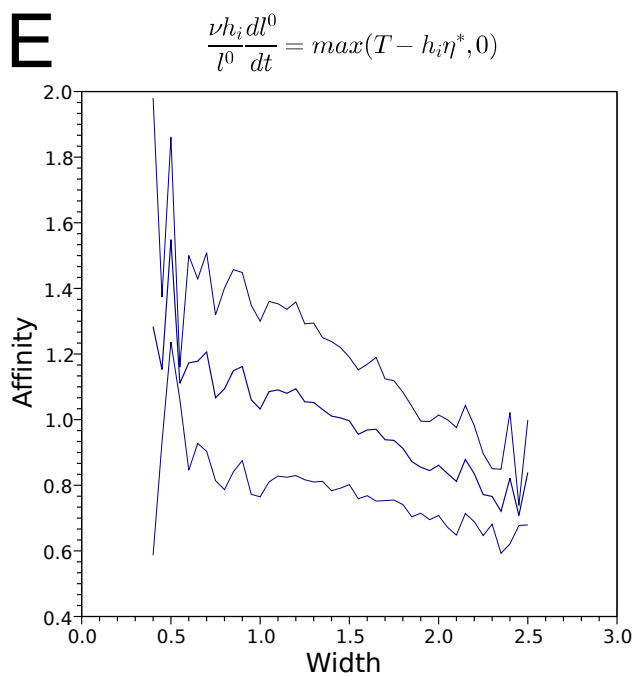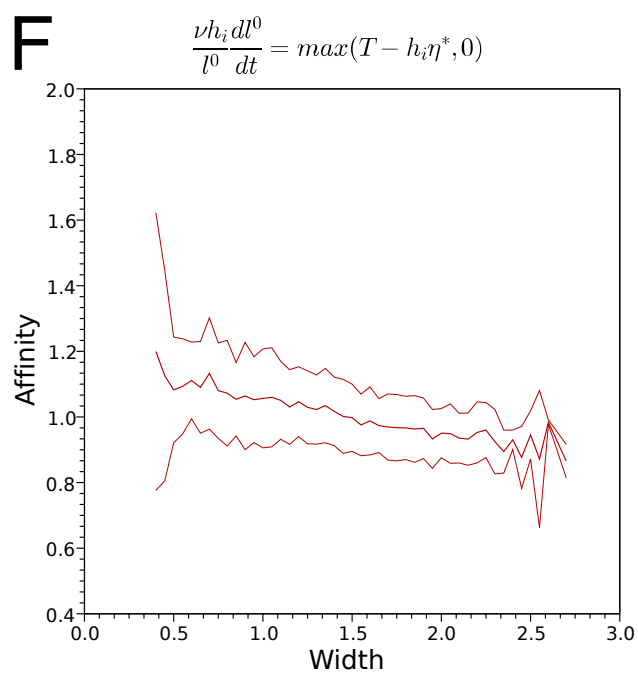

Supplement: S3 Fig — The non-affinity index q of an areole plotted as a function of the average thickness of veins surrounding the areole, without (A,C,E) or with (B,D,F) external stress; the middle line stands for the average value of q over all simulations and the top and bottom lines show the average plus or minus one standard deviation. At the top of each subfigure, the growth law specifies the relative growth rate 1/li0dli0/dt as a function of effective viscosity ν, tension in the vein T, vein thickness hi and law parameter η⋆. (A,B) Linear growth law and no noise. (C,D) Linear growth law and initial noise on vein thickness (amplitude r = 40%). (E,F) Growth law with a threshold and initial noise on vein thickness (amplitude r = 40%). (PDF) [file pcbi.1004819.s003.pdf]

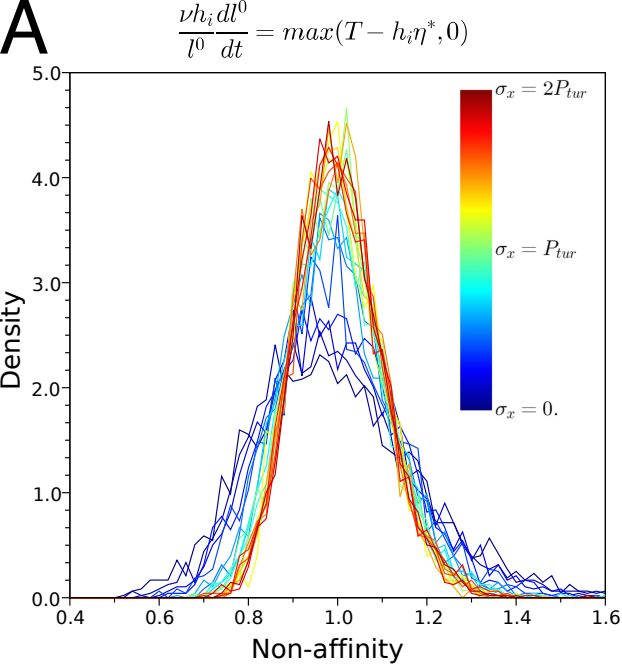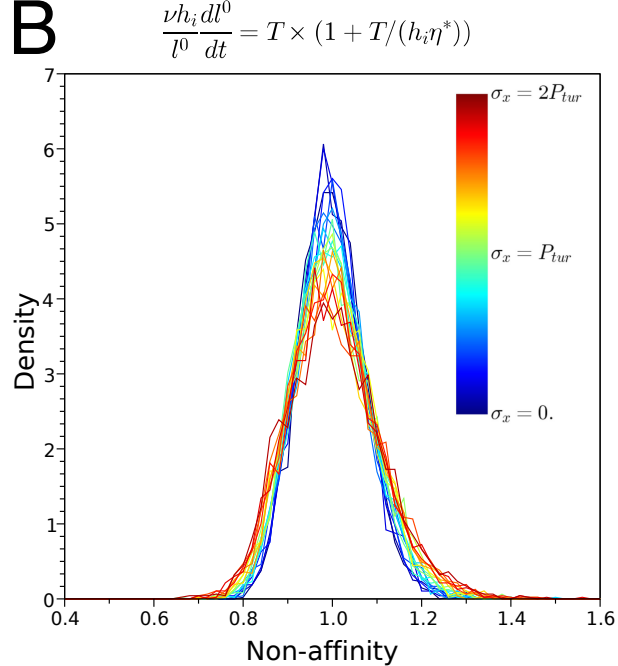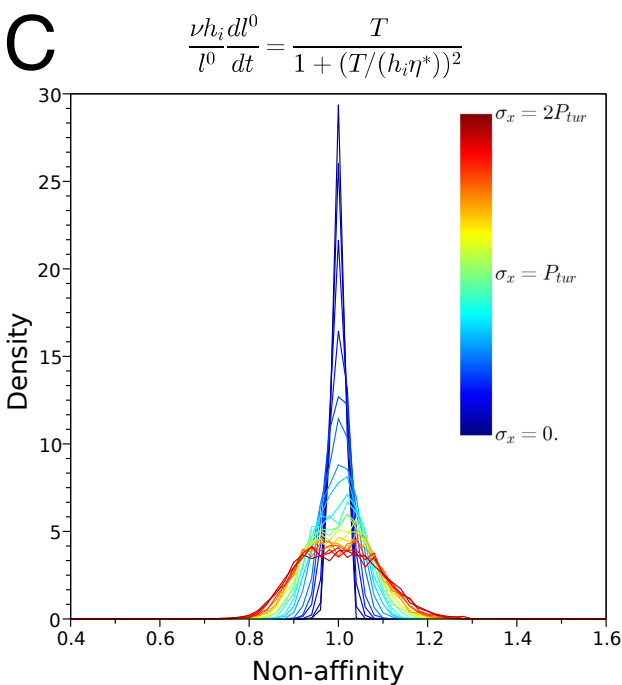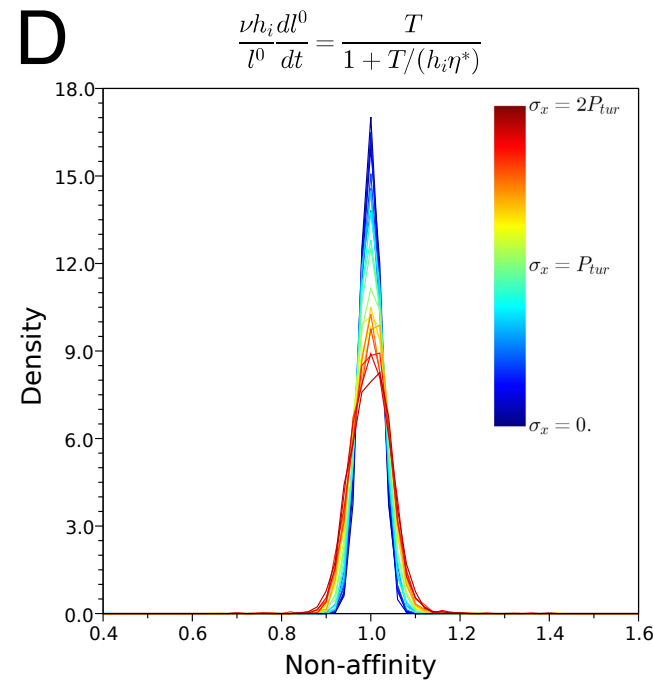

Supplement: S4 Fig — All panels show the stress-dependent distribution of non-affinity index q in all the simulations for a noise amplitude of r = 40% and external stress that increases from σx = 0 (blue) to the maximum value σx = 2Ptur (red). The growth law is shown at the top of each subfigure, specifying the relative growth rate 1/li0dli0/dt as a function of effective viscosity ν, tension in the vein T, vein thickness hi and law parameter η⋆. (A) With a growth threshold. (B) Quadratic. (C) With a maximum. (D) With a saturation. Only the law with a threshold leads to a narrowing of non-affinity under external stress. (PDF) [file pcbi.1004819.s004.pdf]

L1

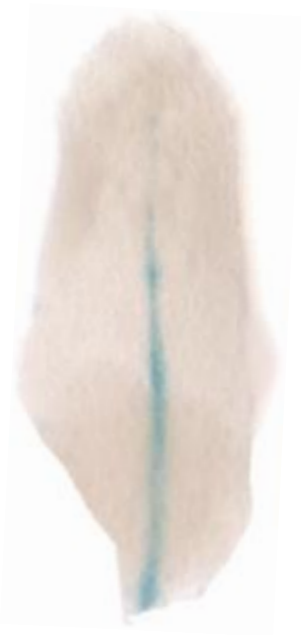

L2

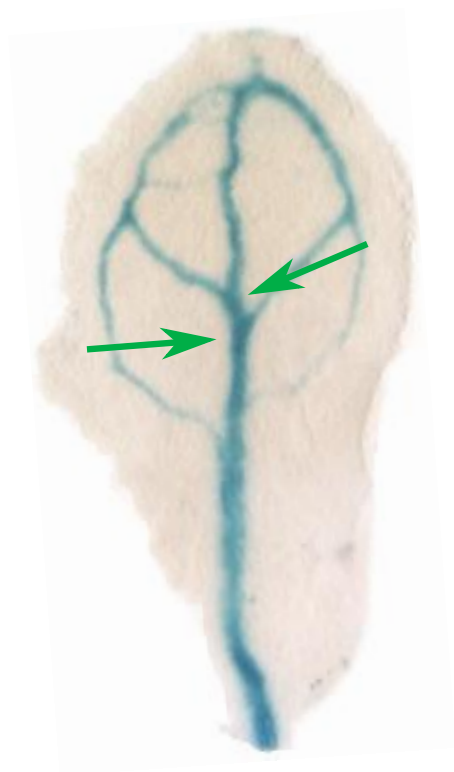

L3

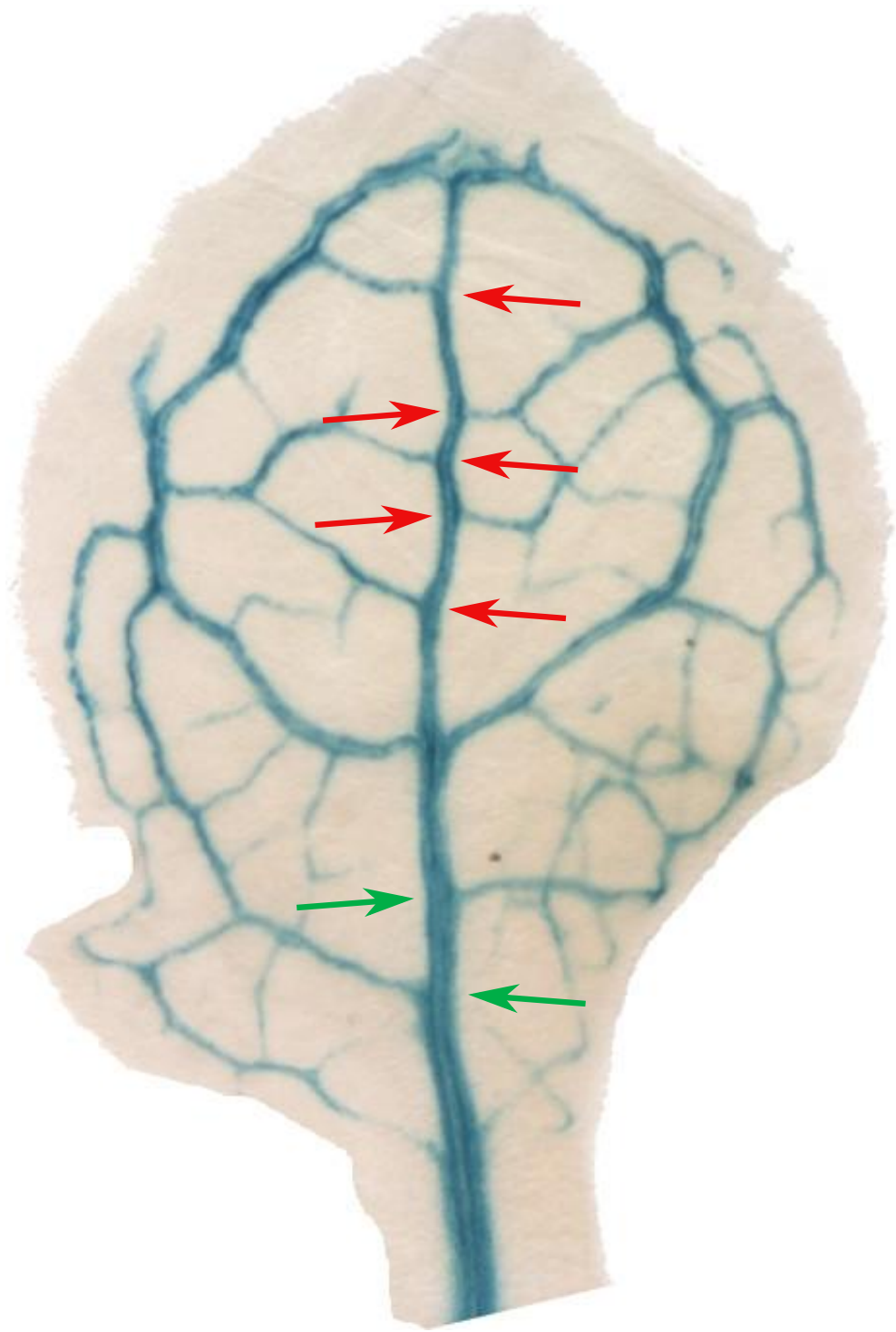

Supplement: S5 Fig — Veins are marked by the early provascular reporter pVH1::GUS. We classify leaves according to whether they have tertiary veins (older leaves, e.g. L3) or not (younger leaves, e.g. L1 and L2). The shape of the midvein in younger leaves is smooth and straight (59 out of 62 leaves) while for older leaves, it is generally kinked at junctions with secondary veins (37 out of 51 leaves). Examples of kinked and unkinked junctions are marked with red and green arrows, respectively. All leaves are at the same scale, L1 being ∼200μm in length. (PDF) [file pcbi.1004819.s005.pdf]

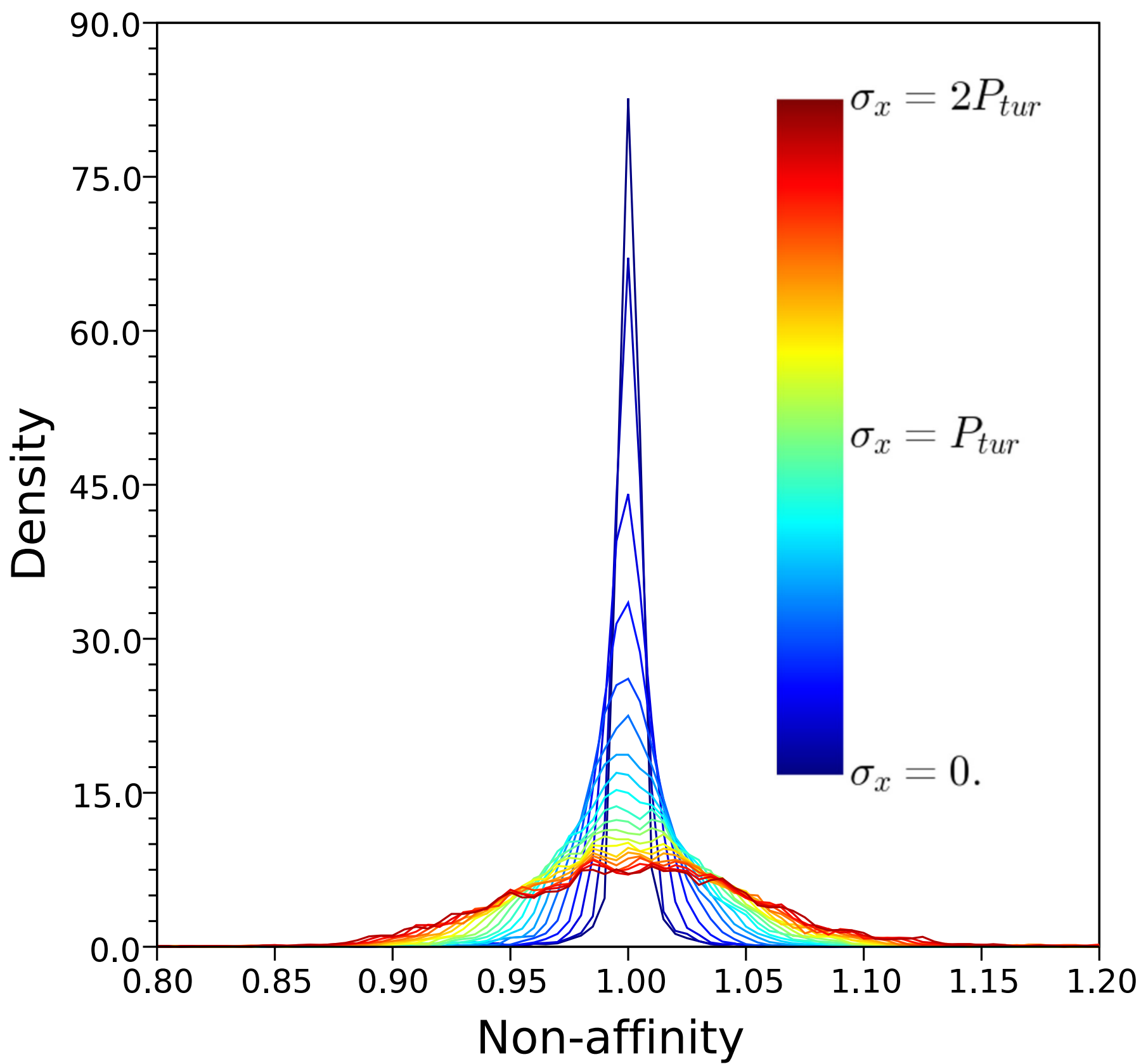

Supplement: S6 Fig — Consider areole i that grows from area Si(t1) at time t1 to Si(t2) at time t2. The local relative areal growth rate is defined as ai = ln[Si(t2)/Si(t1)]/(t2 − t1). The averaged relative areal growth rate is defined from the local one exactly as the averaged texture tensor is defined from the local one. Finally the non-affinity index of areole i is ai/a(r→i). No qualitative changes are seen when replotting Fig 3B using this new definition of the affinity tensor: histograms are shown shown over all realizations with stretching in the x−direction and for a stress σxx ranging from 0 to 2Ptur. (PDF) [file pcbi.1004819.s006.pdf]

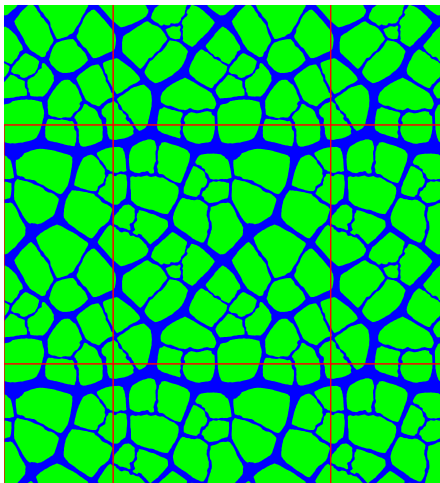

End of vein creation stage.

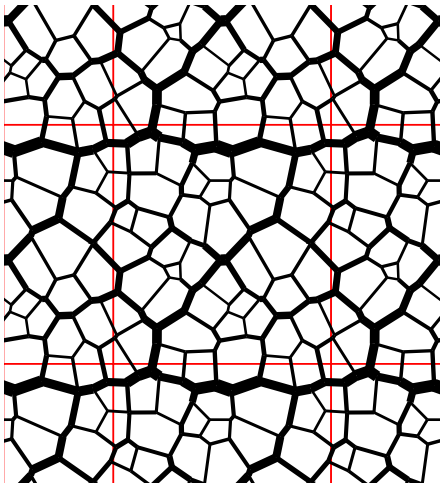

Beginning of vein reorganization stage.

Supplement: S7 Fig — In the left picture each vein and areole contains hundreds of cell walls which are smaller than the resolution of the image. In the right picture each vein is mapped to a viscoelastic rod, and background tissue is completely ignored. (PDF) [file pcbi.1004819.s007.pdf]
